# Safety and efficacy of ketorolac continuous infusion for multimodal analgesia of vaso-occlusive crisis in patients with sickle cell disease

**DOI:** 10.1186/s13023-023-02998-7

**Published:** 2024-01-22

**Authors:** Valeria Maria Pinto, Barbara Gianesin, Salvatore Sardo, Filippo Mazzi, Giammarco Baiardi, Sofia Menotti, Fabio Piras, Sabrina Quintino, Giacomo Robello, Francesca Mattioli, Gabriele Finco, Gian Luca Forni, Lucia De Franceschi

**Affiliations:** 1 EO Ospedali Galliera, Centro della Microcitemia, delle Anemie Congenite e dei Disordini del Metabolismo del Ferro, Genoa, Italy; 2 For Anemia Foundation, Genoa, Italy; 3 Department of Medical Science and Public Health, University of Cagliari, Cagliari, Italy; 4 Department of Medicine, University of Verona and AOUI Verona, Verona, Italy; 5 Department of Internal Medicine, Pharmacology & Toxicology Unit, University of Genoa, Genoa, Italy; 6 Clinical Pharmacology Unit, EO Ospedali Galliera, Genoa, Italy

**Keywords:** Sickle cell disease, Ketorolac, Multi-modal analgesia, Pain

## Abstract

Pain is an hallmark of sickle-cell-related acute clinical manifestations as part of acute vaso-occlusive crisis (VOC). In SCD pain has different origins such as vascular or neuropathic pain, which requires multimodal analgesia. This is based on the administration of drugs with different pharmacological mechanisms of action, maximizing analgesia and minimizing their adverse events and the risk of drug-addition in patients experiencing acute-recurrent pain events as in SCD. Ketorolac is a potent non-narcotic analgesic, being relatively safe and effective during pain-management in children and adults. Up to now, there is a lack of safety information on continuous infusion ketorolac as used to control acute pain in patients with SCD, and the benefits/risks ratio needs to be investigated. Here, we report for the first time the safety profile of ketorolac in the special population of patients with SCD. We confirmed that ketorolac in combination with tramadol, an opioid like molecule, is effective in pain control of adult patients with SCD experiencing acute severe VOCs defined by pain visual analog scale. Our study shows that short term (72 h) continuous infusion of ketorolac plus tramadol is not associated with adverse events such as liver or kidney acute disfunction or abnormalities in coagulation parameters during patients’ hospitalization and within 30 days after patients discharge. This is extremely important for patients with SCD, who should have access to multimodal therapy to control recurrent acute pain crisis in order to limit central sensitization a fearsome issue of undertreated recurrent acute pain and of chronic pain.

**Supplementary Information:**

The online version contains supplementary material available at 10.1186/s13023-023-02998-7.

## To the editor

Sickle cell disease (SCD ORPHA232; OMIM 603,903) is a rare hereditary red cell disorder due to a single-point mutation in the β-globin chain, resulting in the synthesis of pathological hemoglobin S (HbS). Pain is a hallmark symptom of SCD and characterizes sickle cell-related acute vaso-occlusive crisis (VOC). This requires early identification and aggressive medical treatment, making patients with SCD high utilizers of emergency departments (EDs) compared to other severe hemoglobinopathies such as transfusion-dependent thalassemias [1]. The ED management of sickle cell-related VOCs consists of intravenous hydration and pain control with different molecules such as non-steroidal anti-inflammatory drugs (NSAIDs) and opioids. The Scientific Italian Society for the study of Thalassemias and Hemoglobinopathies (SITE) has developed an algorithm to support ED healthcare professionals in the treatment of acute pain in patients with SCD [2]. The innovative aspects of SITE algorithm are the optimization of the triage and of the pain management with multimodal analgesia. This is based on the administration of drugs with different pharmacological mechanisms of action and maximizing analgesia and minimizing their adverse events [3–5]. Additionally, multimodal analgesia controls pain of different origin such as vascular or neuropathic pain that characterizes SCD [6] and decreases the opioid induced post-synaptic changes underlying tolerance and hyperalgesia [7], contributing to the reduction in the incidence of most severe SCD-related complications such as the acute chest syndrome [8].

Although morphine is the most widely used drug for pain management in emergency departments, the parenteral route of administration, dosage, and the recurrency of the treatment may result in long-term drug addiction [9, 10]. In fact, studies in young adults with SCD have shown an increased risk of drug addiction related to undertreated acute pain, which might also increase the risk of chronic pain. Among NSAIDs, Ibuprofen and ketorolac are the most used for treating acute sickle cell-related pain. NSAIDs act at peripheral and central pain-mediated sites that are not involved in the opioid-mu receptor system pathways. When administered in conjunction with opioids such as morphine, NSAIDs are believed to have opioid-sparing effects, therefore, reducing the incidence of adverse events (e.g.: nausea/vomiting) in patients with acute, post-operative pain. However, studies assessing the clinical efficacy of NSAIDs in patients with SCD are limited and their use in SCD is based on expert opinion and clinical experience. Ketorolac is generally offered first to patients with moderate to severe pain because it is a potent non-narcotic analgesic with few adverse effects and has been shown to have a narcotic-sparing. Ketorolac has been shown to be relatively safe and effective during pain management in children and adults with estimated a 1% incidence of hepatotoxicity and nephrotoxicity, including acute and chronic kidney failure in the hospital setting [11–13]. There is a lack of safety information exists on continuous infusion ketorolac use in SCD patient population, and the benefits/risks ratio of its administration during pain crises requires further evaluation.

Here, we report a retrospective study on the safety of infusion ketorolac, as part of multimodal analgesia in adult patients with SCD.

Data were collected from the clinical records of patients with SCD over a decade period (2011–2021) at two tertiary centers (Verona and Genoa). Patient privacy was protected through pseudonymization; therefore, this study was considered exempt from a formal evaluation by the Institutional Review Board. We analyzed SCD patients older than 18 years with severe painful VOCs, defined as bone pain (i.e.: extremities, hips or back) or abdominal pain, for which no other clinical explanation was identified, with a Visual Analogue Scale pain level (VAS) of 70 mm or more. Each hospitalization was defined as an episode of VOC [5]. The multimodal analgesia protocol consisted of a continuous infusion of ketorolac (0.86 mg/Kg/day), tramadol (patient’s weight ≤ 55 kg: 7.2 mg/Kg/day; patient’s weight > 55 kg: 400 mg/die), and metoclopramide (0.57 mg/Kg/day) in normal saline at an infusion rate by weight with an around the clock administration of a proton pump inhibitor as previously described for a maximum of 72 h [5]. The multimodal analgesia was early terminated whenever the pain-VAS was stably lower or equal to 3 in at least two sapare, sequential determination at 3 h interval. Laboratory parameters from the baseline assessment, i.e., the last measurement available before the VOCs, the day of admission, the subsequent 6 days, and at the follow-up of 15 and 30 days were collected (see statistical methods in supplementary data). Data on pain VAS were collected at patient admission and at 72 h after the infusion of multimodal analgesia.

We included thirty-one patients (n = 31) in the analysis (38.7% males, age 33 ± 10 years) for a total of 48 events (Table 1). The genotype distribution was predominantly of type SS with 20/31 patients (64.5%), and the remaining were Sβ (9/31, 29.0%) and SC (2/31, 6.5%). The mean duration of ketorolac therapy was 2.6 ± 1.5 days. In 26/48 (54%) of the cases, patients had already assumed analgesic therapy in the three days before starting infusion of ketorolac (n = 17 for single, n = 6 for double, and n = 3 for triple therapy). In particular, patients used as self-medication: (i) paracetamol (1 gr three times a day) or tramadol (100 mg twice a day); (ii) paracetamol (1 gr three times a day) associated with NSAID such as ketoprofen (80 mg twice a day) or diclofenac as intramuscular injection (75 mg twice a day);paracetamol/codeine (500/30 mg twice a day), or codeine (30 mg four times a day)/ketorolac (10 mg three times a day); (iii) ibuprofen/tramadol/paracetamol (400 mg twice a day/100 mg twice a day/1 gr three times a day). None of the patients were on either chronic analgesic treatment or on anti-coagulant therapy. No major bleeding was observed during the monitoring period. Consistent with our previous reports, we found a significant reduction in pain-VAS at 72 h after infusion with multimodal analgesic therapy (mean reduction 7, 95% CI: 6.3–7.7, p < 0.001) [12, 13]. In general, we observed a mean decrease in hemoglobin value of 1.03 g/dl (95% CI: 0.72–1.35 g/dl, p < 0.001) during the acute phase of VOCs in comparison to the historical value for each patient. This is expected since acute VOCs are generally associated with a mild to severe worsening of hemolysis, even if change in LDH did not each significance in our patient cohort. No significant difference in reticulocyte count was observed (p = 0.21). Parenteral ketorolac administration is licensed for an administration of 2 days and as an enteral formulation for 5 days, since post-marketing surveillance showed a sharp increase in the incidence of upper gastro-intestinal bleeding and acute kidney injury after 5 days [11, 14, 15]. No statistically significant difference in creatinine and eGFR were found between baseline, day 1 and the following time points. This was confirmed by the multivariate analysis, which did not show a significant dependence of creatinine on time, while only gender was associated with creatinine values (p = 0.013), indicating that male patients had higher values of creatinine. This difference was also observed at basal assessment, with a value of 0.79 mg/dL (95% CI: 0.69–0.88) in males and of 0.60 mg/dL (95% CI: 0.53–0.67) in females (p = 0.0012). However, no intra-gender difference for eGFR was observed. Among the comparisons of laboratory parameters at basal, day 1, final evaluation, and at follow-up at 15 and 30 days after patients’ discharge, a statistically significant difference was observed in AST values between the basal and the first day of evaluation (p = 0.045) and in the aPPT ratio between day 1 and the final observation (p = 0.024) (supplemental table 1S). Noteworthy, this difference was found in the subgroup of patients who did not take NSAIDs or paracetamol treatment at home in the 48 h before the access to the hospital for acute VOCs (supplemental table 2S). Therefore, we suggest that our analgesia was not involved in this hemostatic alteration since both PT and aPTT prolongation have been described during VOCs (17).

**Table 1:** Characteristics of the patients and of sickle cell related acute events

	All (n = 31)	SS (n = 20)	Sβ (n = 9)	SC (n = 2)
Patients (% males)	31 (38%)	20 (35%)	9 (56%)	2 (0)
Age (yrs.), mean (SD)	33 (10)	31 (10)	39 (10)	31 (6)
Patients with #1 event	19	13	5	1
Patients with #2 events	9	6	2	1
Patients with #3 events	2	1	1	-
Patients with #5 events	1	-	1	-
Splenectomy or functional asplenia (n)	16	10	6	-
Cholecystectomy	14	10	4	-
HU therapy	22	14	7	1
ICT	8	6	2	-
PPI	20	13	6	1
Number of events	48	28	17	3
Days of ketorolac, mean (SD)	2.6 (1.5)	2.8 (1.6)	2.5 (1.5)	1.3 (0.6)
Previous therapy (%)	26 (54%)	14 (50%)	10 (59%)	2 (67%)
Subsequent hospitalizations (%)	15 (31%)	9 (31%)	5 (29%)	1 (33%)
Hemoglobin (g/dl) baseline	10.1 (1.1)	10.0 (1.1)	10.1 (0.8)	12.0 (0.07)
Hemoglobin (g/dl) acute	9.1 (1.4)	8.8 (1.3)	9.4 (1.5)	11.3 (1.4)
Reticulocyte (10^9^/L) baseline	270 (131)	288 (133)	235 (125)	126
Reticulocyte (10^9^/L) acute	323 (178)	309 (131)	408 (295)	129

Yrs: years; SD: standard deviation; ICT: iron chelation therapy; PPI: proton pump inhibitor; HU: hydroxyurea

**Fig. 1:**
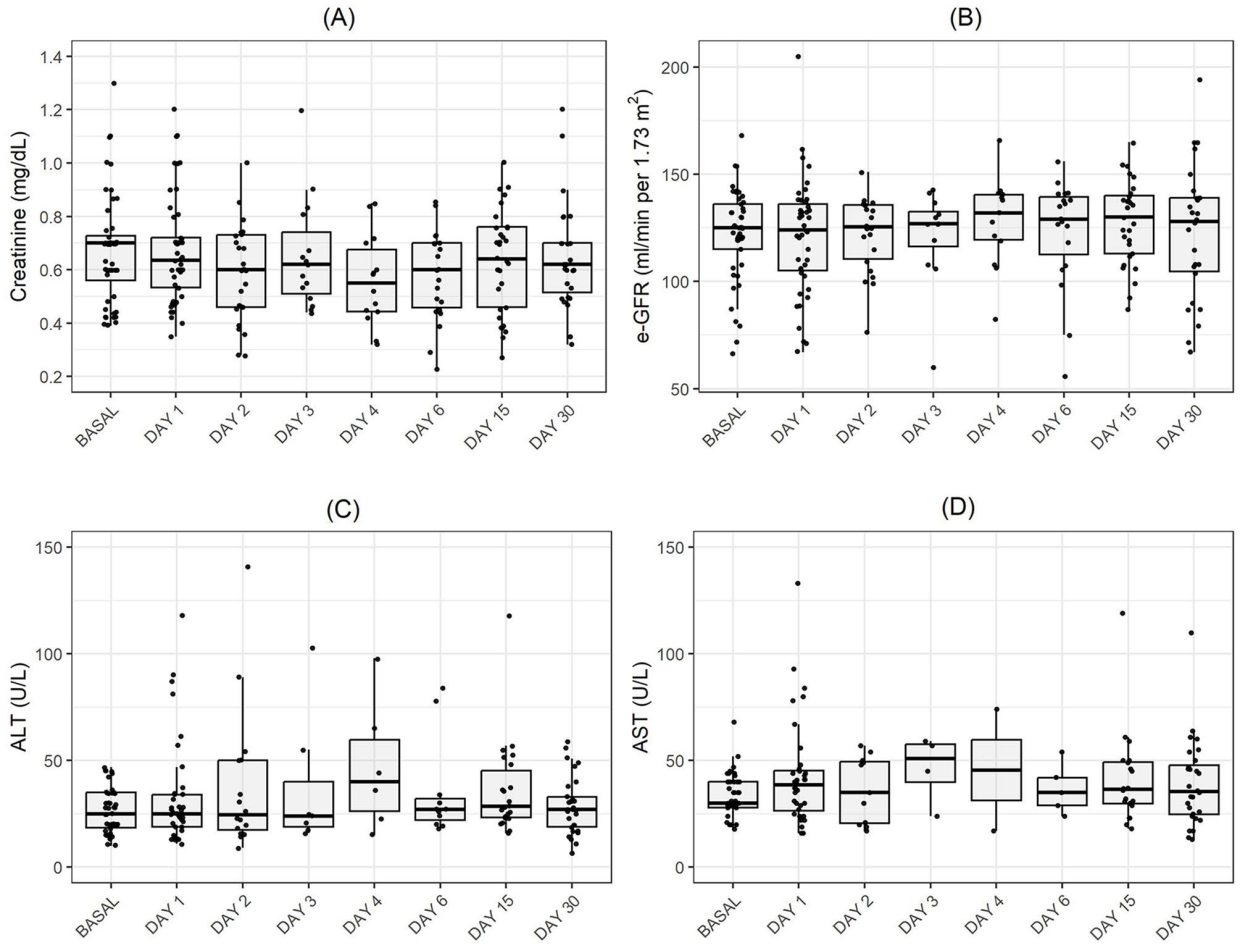
Creatinine (**A**), estimated glomerural fitration rate (e-GFR) (**B**), Alanine amino-trasnferase ALT (**C**) and aspartate amino transferase (AST) (**D**) at basal, during therapy and up to 30 days after multimodal analgesic therapy with ketorolac plus tramadol

Collectively, our data indicate an acceptable safety profile of ketorolac for the clinical management of acute VOCs. We suggest limiting the infusion of ketorolac to the dosage of 0.86 mg/Kg/day for 72 h as part of a multimodal analgesic strategy. This is extremely important for patients with SCD, who should have access to multimodal therapy to control recurrent acute pain crisis in order to limit central sensitization a fearsome issue of undertreated recurrent acute pain and of chronic pain (Fig. 1).

### Electronic supplementary material

Below is the link to the electronic supplementary material.

**Supplementary Material 1:** Additional details on methods, statistics and tables on laboratory results

## Data Availability

The data that support the findings of this study are not openly available due to reasons of sensitivity and are available from the corresponding author upon reasonable request. Data are located in controlled access data storage at E.O.Ospedali Galiera.
