# Supplementary material for: Safety and efficacy of ketorolac continuous infusion for multimodal analgesia of vaso-occlusive crisis in patients with sickle cell disease
Source: Orphanet J Rare Dis. 2024 Jan 22;19:22. doi: 10.1186/s13023-023-02998-7 (PMC10801952; doi:10.1186/s13023-023-02998-7)
Supplement: Supplementary file 1 — Supplementary Material 1: Additional details on methods, statistics and tables on laboratory results [file 13023_2023_2998_MOESM1_ESM.pdf]

Supplementary - Material and Methods

We considered multiple events in the same individual as independent if they were separated in time by at least three weeks.

For all patients, we collected the following laboratory parameters: serum creatinine, estimated glomerular filtration rate (e-GFR), alanine (ALT), aspartate aminotransferase (AST), prothrombin time as international normalized ratio (INR), activated partial thromboplastin (aPTT) ratio, and lactate dehydrogenase (LDH). We included laboratory values from the baseline assessment, i.e., the last measurement available before the vaso-occlusive crisis, the day of admission, the subsequent 6 days, and at the follow-up of 15 and 30 days.

We presented continuous variables as the mean ± standard deviation (SD). We performed paired comparisons between days with the parametric t-test or the non-parametric Wilcoxon signed-rank test in accordance with the normality of the distribution, which was tested with the Shapiro-Wilk test. The Benjamini and Hochberg correction was used to adjust for multiple comparisons.

We managed the missing values of creatinine in the first week by performing a linear interpolation between two consecutive creatinine values. In order to explore any variation of creatinine values that could relate to ketorolac infusion, we carried out multiple pairwise comparisons of creatinine values measured during ketorolac therapy, using as reference values both the day of admission and the baseline values.

We extended the comparisons of creatinine, eGFR, ALT, and AST up to the follow-up values at 15 and 30 days.

For all the other measured parameters, the last evaluation was compared with the ones of the first day and the baseline.

Furthermore, we built a mixed linear model[1] to study the dependence of creatinine on time (within the first 6 days), gender, age, duration of ketorolac administration, and variation of hemoglobin between the admission and the most recent stable value.

A p value of 0.05 was taken as the level of statistical significance. All analyses were conducted using R (version 4.22)[2].

1. Bates D, Mächler M, Bolker B, Walker S (2015). "Fitting Linear Mixed-Effects Models Using lme4." *Journal of Statistical Software*, 67(1), 1–48. doi:10.18637/jss.v067.i01

2. R Core Team (2022). R: A language and environment for statistical computing. R Foundation for Statistical Computing, Vienna, Austria. URL https://www.R-project.org/.

Supplementaty Table 1 Laboratory parameters

	INR		APTT		LDH		FBG	
	Mean (SD)	N	Mean (SD)	N	Mean (SD)	N	Mean (SD)	N
**Basal**	1.12 (0.08)	34	0.99 (0.11)	32	456 (300)	35		
**Day 1**	1.13 (0.08)	26	0.96 (0.11)	25	523 (433)	25	64 (133)	26
**Final evaluation**	1.15 (0.09)	31	1.06 (0.19)	30	435 (346)	32	71(199)	29
**day 1 vs basal**	p=0.8	25	p=0.057	24	p=0.22	22		
**day 1 vs final**	p=0.34	22	p=0.024	22	p=0.22	17	p=0.20	22

Supplementaty Table 2 Comparison of patients with and without previous analgesic therapy

	previous analgesic therapy Yes	***previous analgesic therapy No***	
ALT basal	27.9	23.1	p=0.11
ALT day 1	36.0	42.3	p=0.7
ALT basal vs day 1	p=0.2	p=0.2	
ALT day 1 vs fin	P=0.2	p=0.48	
AST basal	34	32	p=0.59
AST day 1	48	58	p=0.65
AST basal vs day 1	p=0.15	p=0.19	
AST day 1 vs fin	p=0.15	p=0.56	
APTT basal	0.96	1.01	p=0.16
APTT day 1	0.97	0.94	p=0.49
APTT basal vs day 1	p=0.75	p=0.07	
APTT day1 vs fin	p =0.25	p=0.023	
